# A Case of Fournier's Gangrene Secondary to Varicella Zoster Virus in a 7-Year-Old Boy

**DOI:** 10.1155/cris/6094099

**Published:** 2025-01-07

**Authors:** Eimear Phoenix, Eoin O'Broin

**Affiliations:** ^1^Department of Plastic and Reconstructive Surgery, Cork University Hospital, Cork, County Cork, Ireland; ^2^Department of Surgery, Royal College of Surgeons, Dublin, County Dublin, Ireland

## Abstract

A rare case of Fournier's gangrene (FG) secondary to varicella-zoster virus (VZV) affecting the penis and scrotum of a 7-year-old boy is presented. To the authors' knowledge, there are four cases of FG in children as a result of VZV reported to date. Our patient underwent a total of four surgical debridements and was reconstructed using a split-thickness skin graft (SSG). At 1-year post-reconstruction, his graft is robust, and he has no functional issues.

## 1. Introduction

Fournier's gangrene (FG) is a rapidly progressive necrotising fasciitis of the external genitalia and perineal area with sequelae of gangrenous destruction and systemic instability [1]. It is a rare but life-threatening urological emergency more common in the adult population, with a peak incidence between the 2nd and 5th decade [1]. Paediatric cases are exceptionally rare, with less than 60 cases reported in the literature to date, the majority of these presenting within the first 3 months of life [2, 3]. FG in children secondary to varicella-zoster virus (VZV) is even more infrequent, with four cases reported in the literature to date [4–6].

## 2. Case Presentation

**Figure 1:**
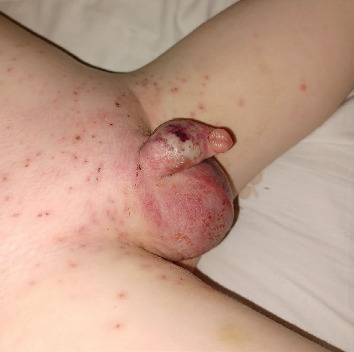
Clinical image obtained on presentation to the emergency department, showing compromised skin of the external genitalia and widespread vesicles.

A 7-year-old boy with a 4-day history of VZV presented to our emergency department with worsening scrotal pain and oedema. His mother reported a deterioration in his condition 48 h prior, with vesicles rapidly spreading across the left scrotum and perineal area. Examination revealed compromised tissue over the entirety of the left scrotum and penile shaft (Figure 1). His inflammatory markers were elevated; white cell count 12.8 and CRP 327. Aside from one isolated episode of pyrexia 24 h prior to this presentation, he remained haemodynamically stable.

He was commenced on broad-spectrum antibiotics (piperacillin/tazobactam) and was immediately transferred to the theatre for urgent debridement. This involved near circumferential debridement of the penile shaft, with preservation of the foreskin and a 1 cm ventral skin bridge only. Non-viable tissue overlying the left scrotum was debrided to healthy transversus vaginalis.

**Figure 2:**
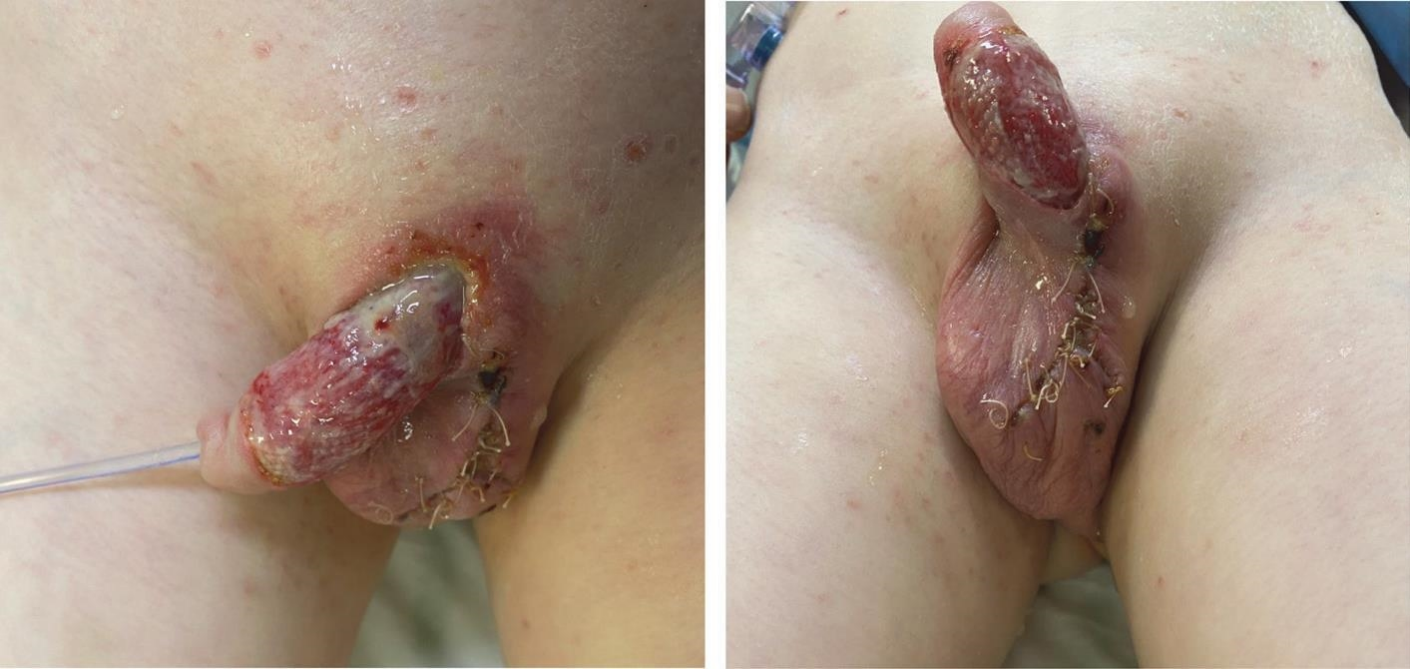
Intra-operative image 48 h following 2nd debridement + closure of the scrotum.

He returned to the theatre 24 h later and underwent further debridement and delayed primary closure of the scrotum (Figure 2). He required a total of four surgical debridements before a healthy granulating wound bed could be established. Scrotal tissue samples from the first debridement cultured staphylococcus aureus sensitive to amoxicillin, and his antibiotics were rationalised accordingly.

**Figure 3:**
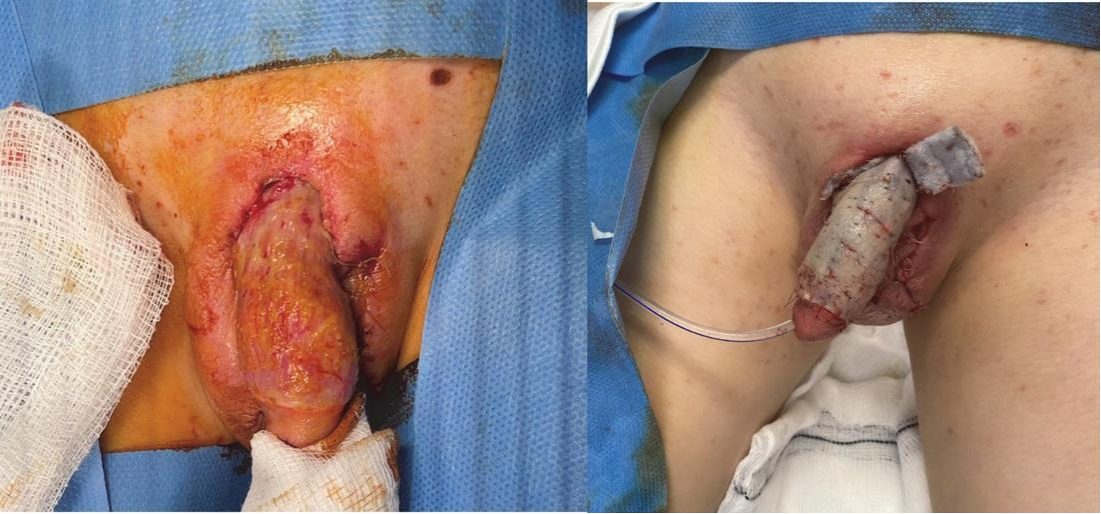
Intra-operative images before and after 4th debridement and SSG reconstruction, respectively. SSG, split-thickness skin graft.

On day 7 of admission, he underwent reconstruction of the penile shaft using a split-thickness skin graft (SSG). A thick SSG was harvested from the left anterolateral thigh, fenestrated and inset with ARTISS and 6.0 Vicryl Rapide (Figure 3). This was secured with a “donut” dressing constructed of Jelonet and circumferential foam secured with 4.0 nylon sutures. His overall condition remained stable, and his inflammatory markers continued to a downtrend.

**Figure 4:**
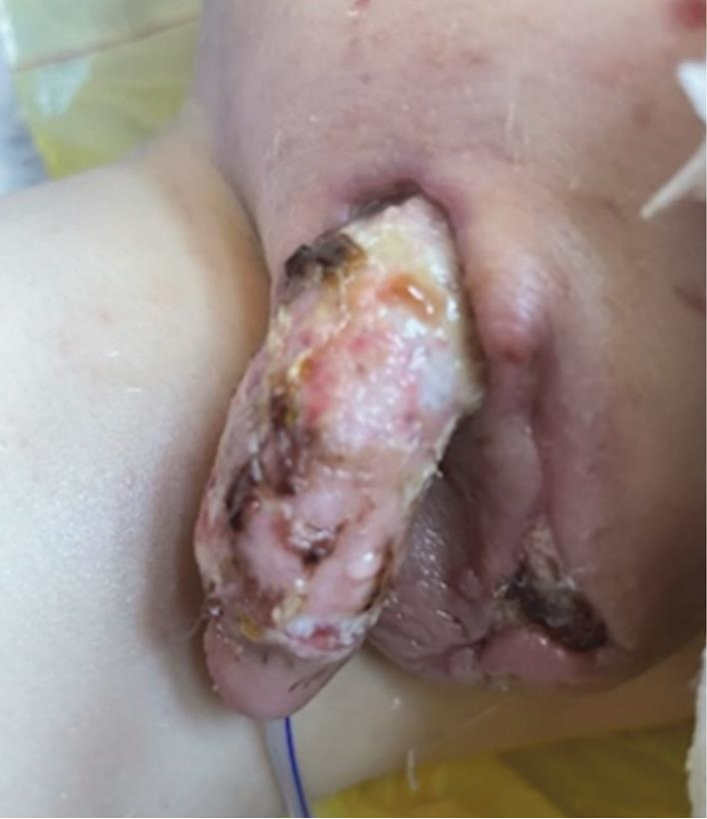
Graft check post-operative day 5 demonstrates 80% graft take.

A graft check was performed on post-operative day 5, and 80% graft take was achieved without evidence of infection or further necrosis (Figure 4). Following advice from our microbiology colleagues, his IV antibiotics were converted to oral form, and he completed a 4-week course in total.

**Figure 5:**
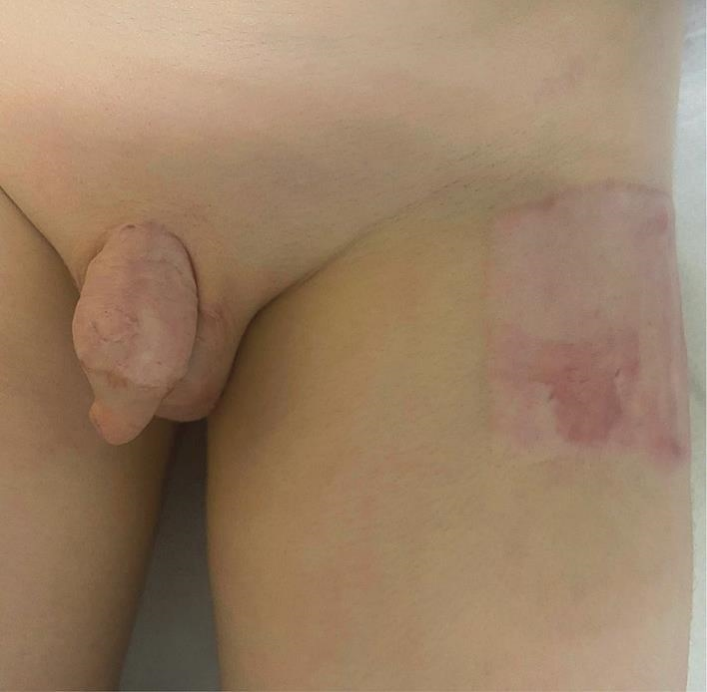
Graft and donor site 12 months post-operatively.

At 12 months post-operatively, his skin graft remains robust with no evidence of early contracture or adverse scaring (Figure 5). His donor site is fully healed and maturing as expected. He remains under regular review in our outpatient clinic.

## 3. Discussion

FG is a necrotising fasciitis of the perineal area, which can rapidly progress to involve the scrotum and penis. FG is exceptionally rare in the paediatric population, with cases secondary to VZC even more unusual. Host susceptibility factors vary between adult and paediatric populations, with the former commonly suffering co-morbidities that render them immunocompromised [1]. Contrary to this, affected children are typically healthy. In paediatric cases, the necrotising process commonly derives from an infection in the urogenital region and/or skin of the external genitalia or perineum [2, 3]. Other aetiologies include preceding trauma, circumcision, anorectal conditions and systemic infection. Mortality rates can reach 50% and may result from sequelae of septicaemia, coagulopathy and acute renal failure [2, 3].

FG is a true surgical emergency requiring timely diagnosis and intervention. Urgent surgical debridement is the mainstay of treatment, accompanied by broad-spectrum antibiotics and immediate resuscitation if required. During debridement, it is important to exercise caution when manipulating the penis and scrotum to prevent triggering systemic sepsis, rare sequelae in adults but also possible in the paediatric population, particularly in the setting of fragile VZV papules [7]. Adequate debridement of necrotic tissue is paramount to ensure source control [1, 2]. FG is a polymicrobial infection, and adequate tissue samples for culture and sensitivities are imperative to target antibiotic therapy [1, 2]. Following sufficient debridement, closure or reconstruction can only be considered when a healthy wound bed free of infection has been established.

VZV is a self-limiting infectious virus arising in children typically between 2 and 8 years of age [5]. To the authors' knowledge, there are only four previous cases of FG secondary to varicella reported in the literature [4–6]. In such cases, FG is thought to arise due to inoculation of the skin vesicles with a secondary bacteria. All four children were healthy, with no significant medical co-morbidities.

Various reconstructive options have been reported in the literature following the debridement of FG. Jefferies, Saw and Jones [6] report a case of a 5-year-old boy who suffered FG of his entire scrotum secondary to VZV. This required two surgical debridements, 10 days of IV antibiotics and was reconstructed with a muscle flap and FTSG. Kalayci et al. [8] reconstructed a perianal and peri-scrotal defect secondary to FG using an SSG and reported a satisfactory outcome. Other reconstructive options include dermal matrixes, myocutaneous flaps or axial groin flaps.

FG is a life-threatening condition requiring immediate attention with combined input from multiple specialties to ensure timely diagnosis and management and increase the chances of a favourable outcome.

## 4. Conclusion

FG is a rare occurrence in the paediatric patient. It carries high morbidity and mortality both from the disease itself and the reconstructive sequelae. Awareness of this condition is needed in children presenting to the emergency department with acute perineal symptoms with a recent history of VZV. Timely recognition and surgical debridement are essential for a successful outcome.

## Data Availability

The authors have nothing to report.
